# Application of Path Planning and Obstacle Avoidance for Riverbank Inspection

**DOI:** 10.3390/s23229253

**Published:** 2023-11-17

**Authors:** Jhong-Wei Jhang, Jih-Gau Juang

**Affiliations:** Department of Communications, Navigation and Control Engineering, National Taiwan Ocean University, Keelung 202301, Taiwan; will_chang@unimicron.com

**Keywords:** path planning, obstacle avoidance, artificial potential field, improved particle swarm optimization

## Abstract

Most coastal trash comes from land. To prevent and control ocean pollution, it should be handled using sources that can maintain a clean ocean and improve the marine ecological environment. The proposed system can be used to inspect riverbanks and identify garbage on riverbanks. This waste can then be cleaned up before flowing into the sea. In this study, we utilized an unmanned aerial vehicle (UAV) to inspect riverbanks and applied path planning and obstacle avoidance to enhance the efficiency of UAV performance and ensure good adaptability in a complicated environment. Since most rivers in the middle and upper sections of the study area are rough and meandering, path planning was first addressed so that the drone could use the shortest path and less energy to perform the inspection task. Branches frequently protrude from the riverbank on both sides. Therefore, an instant obstacle avoidance algorithm was added to avoid various obstacles. Path planning was based on an Improved Particle Swarm Optimization (IPSO). A fuzzy system was added to the IPSO to adjust the parameters that could shorten the planned path. The Artificial Potential Field (APF) was applied for real-time dynamic obstacle avoidance. The proposed UAV system could be used to perform riverbank inspection successfully.

## 1. Introduction

UAVs were first utilized primarily for military reconnaissance missions or attack operations. In recent years, drones have been mainly used in civilian applications, including aquaculture, material transportation, agricultural spraying, river inspection, rescue work, and live broadcasting. UAVs can now be used to address the shortage of human resources, improve work efficiency, and conduct field surveys in dangerous areas. Drones have brought great convenience to society in many aspects, but many problems must be solved, such as safety. In addition to safety, there are other issues that many researchers have attempted to decipher, including how to make drones arrive at their destination faster, complete work more efficiently, and avoid external threats, among others.

There are three types of UAVs: fixed-wing, helicopters, and multi-rotor. Each has its advantages and disadvantages. Fixed-wing UAVs have longer flight times and faster speeds and require more space for take-off and landing. Although helicopters can take off and land vertically, hover at the same altitude, and have exceptional flexibility, its complex structure makes operation difficult. Compared with the previous two, multi-rotor UAVs have slower flight speeds and lesser flexibility but can perform vertical take-off, landing, and hovering. Its structure is simple, making it convenient for users to set up sensors and to be used for riverbank inspection.

A six-axis UAV was used in this study. Compared with a four-axis UAV, a six-axis UAV is more stable and has a larger battery capacity, giving it longer endurance. The researchers of this study equipped the six-axis drone with a real-time kernel (RTK) four-galaxy positioning chip that is more precise than a global positioning system (GPS). An NVIDIA Jetson Xavier NX was used to calculate paths, avoid obstacles, and issue control commands to the flight control board, Pixhawk. A LiDAR scanner was employed to measure the surrounding objects’ distance and to obtain obstacle avoidance control. Since some rivers are located in relatively remote and isolated places, the environment will be impacted if wastes build up in the middle and upper reaches of the river because they will be challenging to find. Therefore, this study proposes a system to replace human labor for waste detection on river banks. By using the path planning method presented in this research, drones can fly to river locations that are difficult for people to access and conduct inspection activities.

With UAVs’ popularity, drone use is becoming more diverse, expanding its applications in many fields, such as bridge pier detection systems [1], solar panel maintenance [2], crop and weed detection [3], natural gas leakage estimation [4], fire detection [5], and many others. Therefore, this study explored finding the best route and avoiding dangerous conditions when encountering dynamic objects. There are many methods of planning path, such as Genetic Algorithm (GA) [6,7], Ant Colony Algorithm (ACA) [8,9], Rapidly Exploring Random Tree Algorithm (RRT) [10,11], Particle Swarm Optimization (PSO) [12,13], and Artificial Potential Field method (APF) [14,15]. These algorithms have their respective advantages and disadvantages, and numerous studies have been presented to optimize their ability to find paths better and faster [16,17,18,19,20,21]. In the following sections, the computational efficiency of various path planning algorithms will be discussed, and the courses will be compared to determine the most suitable path planning algorithm for this study. A path planning scheme was also proposed based on Improved Particle Swarm Optimization (IPSO) combined with a fuzzy system. The Artificial Potential Field was applied to obstacle avoidance control. The proposed Fuzzy IPSO (FIPSO) can generate smooth paths without sharp turns when the UAV encounters obstacles. FIPSO outperforms GA, ACO, RRT, PSO, and IPSO in the length of the planned path and time of computation. The UAV can fly along the path safely and automatically. The proposed system can be used to inspect riverbanks and identify garbage on riverbanks. The identified waste can then be cleaned up before flowing into the ocean, which can prevent and control marine pollution.

## 2. System Description

**Figure 1:**
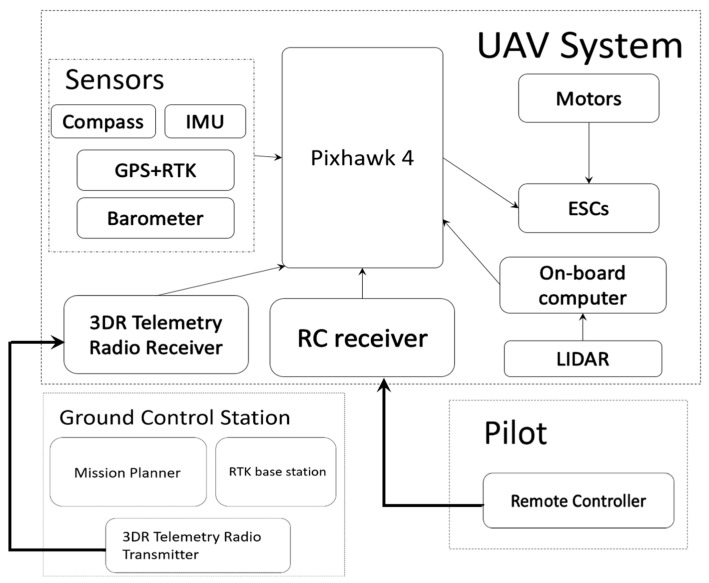
UAV hardware architecture.

The hardware architecture is divided into two parts: the first part is the hexacopter system, and the second is the ground station. The hexacopter system comprises the flight control board, electrical transformer, RC receiver, RTK-F9P, motor, and onboard computer. The operator can control the ground station through a remote control or computer. Combined with the RTK base station, the UAV can obtain the coordinate positions more precisely. Figure 1 illustrates the UAV hardware architecture.

**Figure 2:**
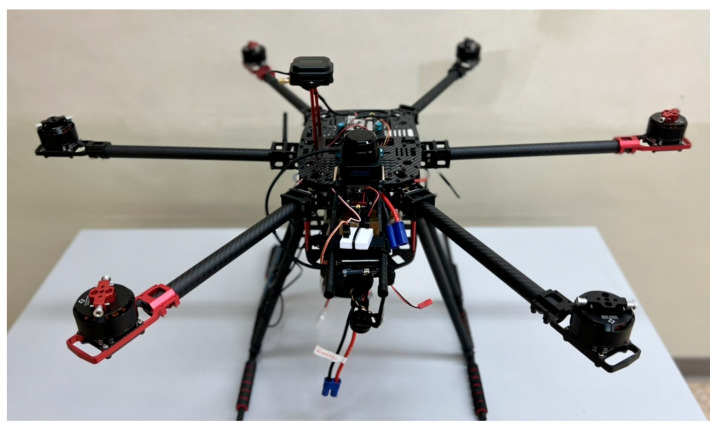
Hexacopter body.

In response to this study, we investigated various UAVs on the market. Multi-rotor UAVs were selected due to their simple structure and small flight space requirement. Following a comparison between four-axis, six-axis, and eight-axis drones, the six-axis drone was more stable than the four-axis drone. In addition, it is lighter and easier to transport than an eight-axis drone. Figure 2 shows the chosen six-axis unmanned aerial vehicle.

The UAV system must determine the ideal path and communicate this information to the flight controller. Since the task requires extensive calculation in the control process and monitoring of various UAV information, an NVIDIA Jetson Xavier NX was chosen [22] as the onboard computer. An NVIDIA Jetson Xavier NX (NVIDIA, Santa Clara, CA, USA) has many desirable features, such as its small size, lightweight, and fast computing speed. It can also deliver up to 21 trillion operations, producing excellent path planning performance.

Drone automation can be achieved when used in conjunction with peripheral equipment. Most outdoor obstacle avoidance uses ultrasonic modules as sensors because sunlight often interferes with radar and LiDAR in the outdoor environment. However, the chosen RPLIDAR-S1 can function effectively even in an outdoor setting. The measurement distance can reach up to 40 m indoors and 30 m outdoors. A 360-degree detection can also be performed, which is highly appropriate for the task involved in this research.

## 3. Path Planning

The proposed system was designed to perform cruise tasks along the predetermined cruise points in response to river cruises. The first part of this study focuses on finding the best path, enabling the UAV cruise to use the shortest route, and improving the UAV’s work efficiency. The second part is more concerned with how the drone should respond if it encounters an unknown obstacle on the way to the cruise mission. The environment can generally be divided into two categories when the UAV performs path planning. The first category is the “known environment”. Regardless of whether the obstacle is stationary or dynamic, the path that can bypass obstacles can be estimated beforehand. The second category is the “unknown environment”. In this category, the UAV can generate a path through real-time estimation to bypass the obstacles it encounters. The Genetic Algorithm, Ant Colony Algorithm, Rapidly Exploring Random Tree Algorithm, Particle Swarm Optimization, and Artificial Potential Field method are some of the path planning methods available. This study compared these five algorithms to find the most suitable path planning algorithm that meets the research objectives.

(1) Genetic Algorithm

A Genetic Algorithm is a heuristic search algorithm created using biological evolution. It imitates the organisms’ genetic evolution process to find the best solution. The three essential operations of the Genetic Algorithm are selection, crossover, and mutation. Selection is typically used to find the best individuals in the population. Conversely, crossover is the recombination of two individuals to produce the next generation of individuals who inherit their parents’ superior genes to produce offspring with higher fitness. There is a very slim possibility that the mutation will be triggered, keeping the genes diverse and preventing the population’s chromosomes from being too close and convergent to a locally optimal solution. At present, several methods have been proposed to improve the algorithm’s efficiency and convergence speed.

(2) Ant Colony Algorithm

The Ant Colony Algorithm is often used to address the traveling salesman problem. It is a behavior that resembles the way ants navigate through paths to search for food. Initially, the ants randomly explore all directions, leaving gradually weakened pheromones along the road. When the ants cross the road with pheromones left, they decide whether to take this path. The pheromones will become stronger if they follow a particular path. The shorter the route, the less time the ants need to travel to and from their nest and foraging site, and the stronger the pheromones they leave behind, which all other ants will eventually follow to search for food. Although the Ant Colony Algorithm does not aimlessly search for foraging points like real ants do, it still has a high level of randomness, which also results in the possibility that the final path may not be the best.

(3) Rapidly Exploring Random Tree Algorithm

Rapidly Exploring Random Tree Algorithm (RRT) is an algorithm for tree data storage architecture. It effectively explores high-dimensional nonconvex space and is often used to solve the path planning of robots in complex environments. RRT has the advantages of a simple architecture. It can perform large-scale spatial exploration without needing a pre-modeled space. Although RRT has numerous benefits, its randomness leads to some shortcomings in the algorithm, resulting from its failure to consider the spatial path cost when exploring.

(4) Particle Swarm Optimization

Like the Genetic Algorithm, Particle Swarm Optimization (PSO) is an algorithm for solving optimization problems. PSO is updated with the cooperation of each particle, unlike the Genetic Algorithm, which uses genetics to produce the most adaptive offspring. PSO is an algorithm created by observing the bird flocks’ foraging behavior. Each particle represents a bird in the flock. The algorithm was updated primarily to adopt the two following points: decide where to fly based on one’s experience and determine the flight direction based on others’ experience. The behavior of the individual particles is unpredictable, but the groups’ behavior is predictable. Since the particles are focused on finding those that are better than them, they refer to and learn. Therefore, all particles will eventually move in a better direction. This study proposed an improved PSO with a fuzzy system that can modify the PSO’s parameters to adjust the obstacle’s avoidance distance and maximum step size.

(5) Artificial Potential Fields

Artificial Potential Fields (APFs) are often used in robot path planning algorithms. The algorithm considers the target point and the obstacle objects that exert gravitational and repulsive forces on the device. The device will move along the resultant force of these gravitational and repulsive forces. To obtain the path, one needs to find the resultant force of the potential field, which is calculated using the two forces generated inside the potential field: repulsion and attraction.

## 4. Obstacle Avoidance

This study combined a fuzzy system with the Improved Particle Swarm Optimization (IPSO) algorithm to achieve path planning and obstacle avoidance. Since the LiDAR used for obstacle sensing in this study is two-dimensional, the path and obstacle avoidance did not consider the height factor. It only calculated for the path on the X-Y plane. The study referred to the modified and Improved Particle Swarm Optimization algorithm proposed by Zhang et al. [23]. They modified the algorithm in five ways:

1. The initialization of particle swarm;
2. Learning factors;
3. Inertia weight;
4. Updating the rule of the particle velocity;
5. The treatment of the particle crossing the boundary.

This study applied some concepts combining the benefits of IPSO and added a fuzzy system to modify the parameters.

(1) Initialization of Particle Swarm

The initial particle distribution in PSO affects the algorithm’s efficiency. A uniform distribution is usually used in traditional methods to generate the initial position of the particle swarm. The uniform distribution has the advantage of being easy to implement, but it does not allow the particles to surround the optimal solution. To improve the initial distribution of particles, β function distribution was proposed.

(1) $$\beta \left(x\right)=\frac{x^{a-1}{\left(1-x\right)}^{b-1}}{\int_{0}^{1}t^{a-1}{\left(1-t\right)}^{b-1}dt},\;\;0<x<1.$$

In [23], set *a = b* < 1. The *β*(*x*) function will be a symmetrical U shape. The initial positions of the particles are distributed near the boundaries of the search space. A better initialization effect can be obtained when *a = b =* 0.8. The equation below demonstrates how to initialize the particle formula for each dimension, where *M* is defined as the number of particles, [${min}_{j},{max}_{j}$] is the range of the *j*th dimension, and $X_{i}$ is the candidate solution. A beta distribution can generate random numbers using the betarand function. In this formula, i = 1, 2, ⋯, M*,* j = 1, 2, ⋯, M, *l* is the lower bound, and *u* is the upper bound of the random value.

(2) $$X_{i}={min}_{j}+\left({max}_{j}-{min}_{j}\right)\times betarand\left(l,u\right).$$

(2) Learning Factors

Learning factors influence communication between particles. In the initial stages of this study, the researchers aimed the algorithm to focus on the ability to search globally. In the later stages, we wanted the algorithm to focus on local optimization so that it could converge to a globally optimal solution. Therefore, particles must have a strong self-learning ability at the beginning and will weaken as the operation progresses. The particles’ social learning ability slowly changes from the weakest to the strongest. The following formula is designed to dynamically change the state value to balance global search and local development:

(3) $$c_{1}=c_{1,initial}+\frac{c_{1,final}-c_{1,initial}}{\max\_iteration}\times t.$$

(4) $$c_{2}=c_{2,initial}+\frac{c_{2,final}-c_{2,initial}}{\max\_iteration}\times t.$$

Here, $c_{1}$ is a self-learning weight that allows the particles to remember their best position and compare the best position with the current position. $c_{2}$ is the social learning ability to record the best position of the particle swarm and compare the best position with the current position. $c_{1,initial}$ and $c_{2,initial}$ represent the initial values of $c_{1}$ and $c_{2}$, respectively. $c_{1,final}$ and $c_{2,final}$ represent the final values of $c_{1}$ and $c_{2}$, respectively. *t* is the current iteration number. max_⁡iteration represents the maximum iteration number.

(3) Inertia Weight

Y. Shi and R. Eberhart introduced inertia weights into the Particle Swarm Optimizer [24]. The inertia weight is an important parameter for PSO. This parameter influences the PSO’s search capability. A larger inertia weight can improve the PSO’s global search ability, while a smaller inertia weight can enhance the PSO’s local optimization ability. A higher inertia weight is required in the beginning to focus on the global situation, and the inertia weight is gradually reduced as the operation is performed to enhance the local optimization ability.

(5) $$w\left(t\right)=w_{min}+\frac{w_{max}-w_{min}}{\lambda }\gamma \left(\lambda ,1-\frac{t}{\underset{}{\max}iteration}\right).$$

Here, *t* is the current number of iterations, and max_⁡iteration is the maximum iteration number. $w_{max}$ and $w_{min}$ are fixed values that are the maximum and minimum values of the inertia weight, respectively. Typically, the maximum value of inertia weight is set to 0.9, while the minimum value of inertia weight is set to 0.4 [20,21]. *λ* is a random variable greater than 0 and can also be set to a fixed value greater than 0. Combined with the inverse incomplete function *γ* to adjust the inertia weight, the formula is given as follows.

(6) $$\gamma \left(\lambda ,a\right)=\int_{0}^{\lambda }e^{-t}t^{1-a}dt$$

**Figure 3:**
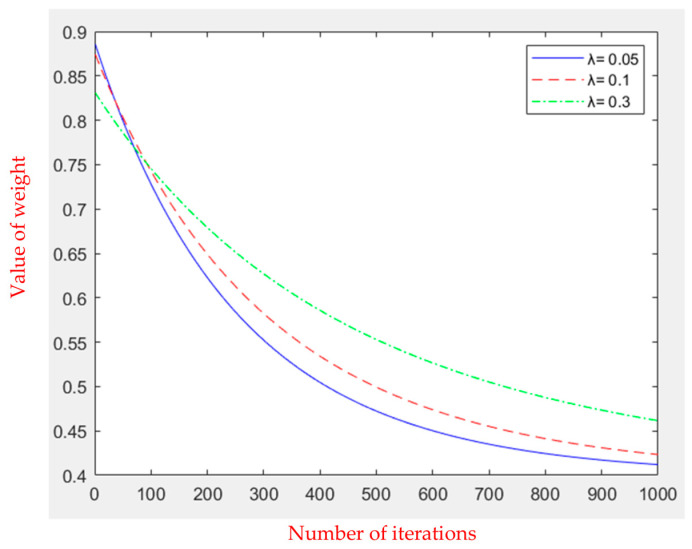
Dynamically declining inertia weights.

The maximum number of iterations is set to 1000; *λ* is set to 0.05, 0.1, and 0.3; and the change in inertia weight is shown in Figure 3. It can be seen that when *λ* is set to 0.05, the inertia weight is larger at the first half of the iteration, and the decreasing speed is faster than the other two. In the second half of the iteration, *λ* = 0.05 is close to the minimum value, strengthening the algorithm’s local optimization ability and effectively avoiding the phenomenon of early convergence.

**Figure 4:**
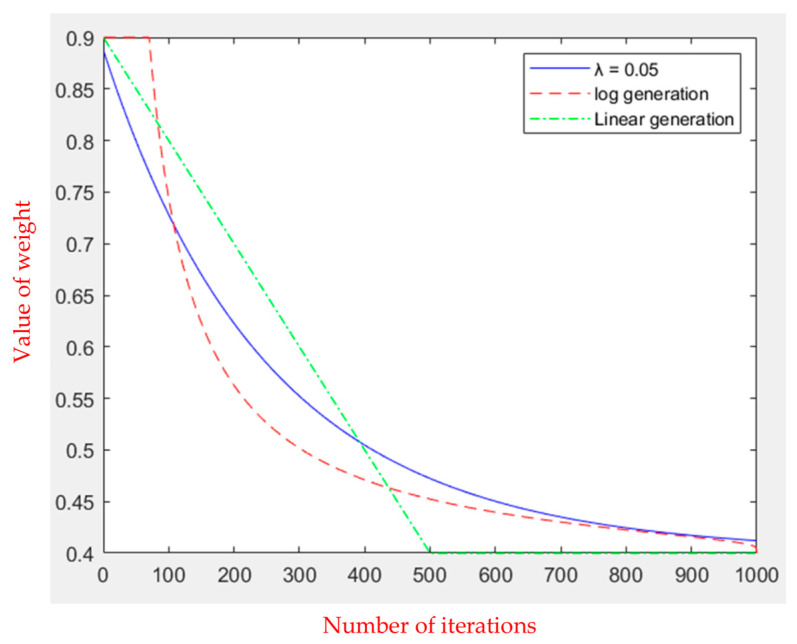
Inertia weights were generated using three different methods.

In this study, two new dynamic change functions of inertia weight are added under the conditions that the maximum number of iterations is 1000, the maximum value of inertia weight is 0.9, and the minimum value is 0.4. First, the logarithm to generate a function for inertial weights was used with the following formula. Second, the first half of the iteration used a linear function to descend to the minimum value, while the second half had the inertia weight equal to the minimum value. Figure 4 [25] depicts the inertia weight changes.

(7) $$w\left(t\right)=w_{min}+{log}_{d}0.93\left(w_{max}-w_{min}\right).$$

*d* is generated using the following formula:

(8) $$d=1-\frac{t}{\max\;iteration}.$$

Figure 4 shows that the inertia weight generated by the logarithm can be kept at its maximum value at the start of the operation and then decrease at a faster rate. Compared with the other two, the linear function decreases the slowest in the early stages of the iteration, but the inertia weight in the later stages is equivalent to the minimum value, which can enhance the local optimization ability. In this study, we compared and added three inertia weight-changing functions to two different environments.

**Figure 5:**
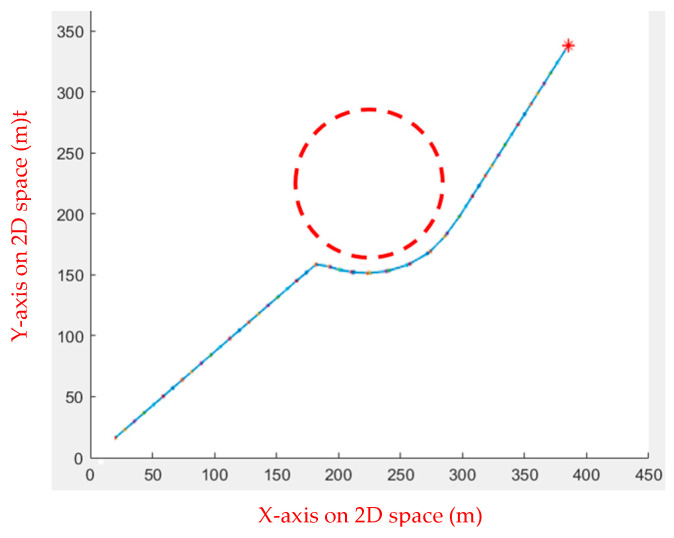
Environment 1: the red circle is the obstacle, the red star is the end point, the blue line is the actual path, and the red dots are the moving steps.

**Figure 6:**
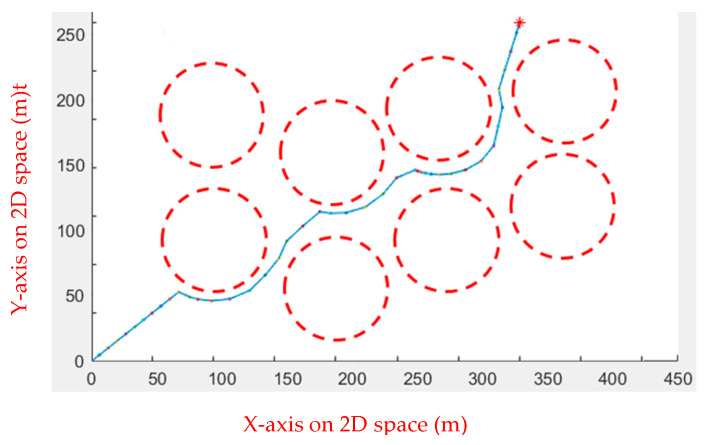
Environment 2: the red circles are the obstacles, the red star is the end point, the blue line is the actual path, and the red dots are the moving steps.

In the first environment, an obstacle with a radius of 45 and a center of (225, 225) was set up to test the algorithm’s performance when dealing with obstacles. As shown in Figure 5, the starting point is at (20, 20), while the ending point is at (350, 350). In the second environment, eight circular obstacles with a radius of 40 were set to test whether the algorithm would fall into a local optimal solution, as shown in Figure 6. Table 1 shows the comparison results of the running speeds. As seen in Table 1, using a linear function to change the inertia weight dynamically outperforms the other two.

(4) Updating the Rule of Particle’s Velocity

To achieve global search and local optimization, a delta function was added to improve the particle velocity update rule, as shown below:

(9) $$v_{i}\left(t+1\right)=w\left(t\right)v_{i}\left(t\right)+c_{1}r_{1}\left(P_{i}^{L}-x_{i}\left(t\right)\right)+c_{2}r_{2}\left(P_{g}^{L}-x_{i}\left(t\right)\right)+\delta_{i}(t).$$

The *δ* formula is as follows:

(10) $$\delta_{i}\left(t\right)={\left(1-\frac{t}{\underset{}{\max}iteration}\right)}^{k}\left(\frac{1}{N}\sum_{i=1}^{N}P_{i}^{L}-X_{i}\right)\times rand.$$

Here, *rand* is a random number in the interval (0, 1), *k* is a fixed value greater than 0, and $\delta_{i}\left(t\right)$ has a strong relationship with *k*. At the beginning of the iteration, the value of ${\left(1-\frac{t}{\max\_iteration}\right)}^{k}$ is large, and so is the value of $\delta_{i}\left(t\right)$, while the value of $\delta_{i}\left(t\right)$ gradually decreases as the iteration increases. $\frac{1}{N}\sum_{i=1}^{N}P_{i}^{L}$ is the average of the particles’ best positions. When $\delta_{i}\left(t\right)$ is large, the ability of the global search can be enhanced, and when $\delta_{i}\left(t\right)$ decreases in the later stage, the ability of local optimization can also be enhanced. This method can significantly improve the balance between global search and local optimization.

(5) Treatment of the Particle Crosses the Boundary

**Figure 7:**
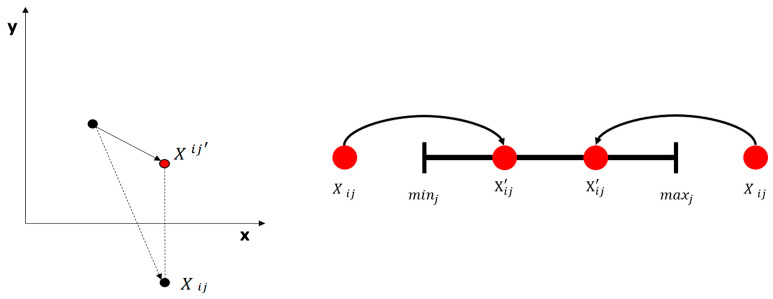
Transboundary treatment method using symmetric mapping.

In PSO, the out-of-bounds processing of the particle position is typically performed by adopting a method wherein the particle position crosses and is equal to the boundary. Although this method is simple and straightforward, there are other better solutions than the boundary position. Therefore, an approach based on symmetric boundary mapping was used, as shown in Figure 7. [${min}_{j},{\;max}_{j}$] is the interval of the j dimension; if the particle position $X_{ij}$ crosses the boundary, it will be treated using the following formula. The particle outside the interval [${min}_{j},{\;max}_{j}$] can be transferred into the interval using Equation (11). If the particle position $X_{ij}$ is greater than ${max}_{j}$, this $X_{ij}$ is guaranteed to be transferred into the interval, and the value is no smaller than ${min}_{j}$. On the contrary, if $X_{ij}$ is less than ${min}_{j}$, $X_{ij}$ is guaranteed to be transferred into the interval, and the value is no more than ${max}_{j}$.

(11) $$X_{ij}^{\prime }=\left\{\begin{array}{c} {\min\;(max}_{j},2{min}_{j}-X_{ij}),\;X_{ij}<{min}_{j}\\ \max\left({min}_{j},2{max}_{j}-X_{ij}\right),\;X_{ij}>{max}_{j} \end{array}\right..$$

(6) Judgment of the Obstacles

Take its own position as the center of the circle, and take ρ as the search radius. If an obstacle between the current position and the next waypoint and the travel route is affected, call the path planning algorithm. Otherwise, go to the next waypoint. The formula for judging obstacles is as follows:

(12) $$\rho >\sqrt{{\left({P_{1}-{obs}_{1})}^{2}+(P_{2}-{obs}_{2}\right)}^{2}}.$$

(13) $$\sqrt{{\left({P_{1}-W_{2})}^{2}+(P_{2}-W_{2}\right)}^{2}}=\sqrt{{\left({P_{1}-{obs}_{1})}^{2}+(P_{2}-{obs}_{2}\right)}^{2}}+\sqrt{{\left({W_{1}-{obs}_{1})}^{2}+(W_{2}-{obs}_{2}\right)}^{2}}.$$

Assume the UAV’s current position is ($P_{1}$, $P_{2}$), the position of the obstacle is (${obs}_{1}$, ${obs}_{2}$), and the position of the next waypoint is ($W_{1}$, $W_{2}$). Equation (12) is used to judge whether obstacles exist within the search radius ρ, whereas Equation (13) is used to determine whether obstacles exist between the current position and the next waypoint position.

The LiDAR used in this study can detect obstacles at a longer distance, and we used path planning to find the shortest path in a known obstacle environment. Since the obstacles are known, the study calculated whether the obstacles would hinder the UAV from its starting position and set two criteria to judge.

1. The angle between the obstacle and the target point is smaller than the angle between the obstacle and the tangent.

(14) $$\theta_{obs,target}<\theta_{obs,tangent}.$$

2. The distance between the starting point and the target point is greater than the distance between the starting point and the center of the obstacle.

(15) $$d_{start,target}>d_{start,obs}.$$

**Figure 8:**
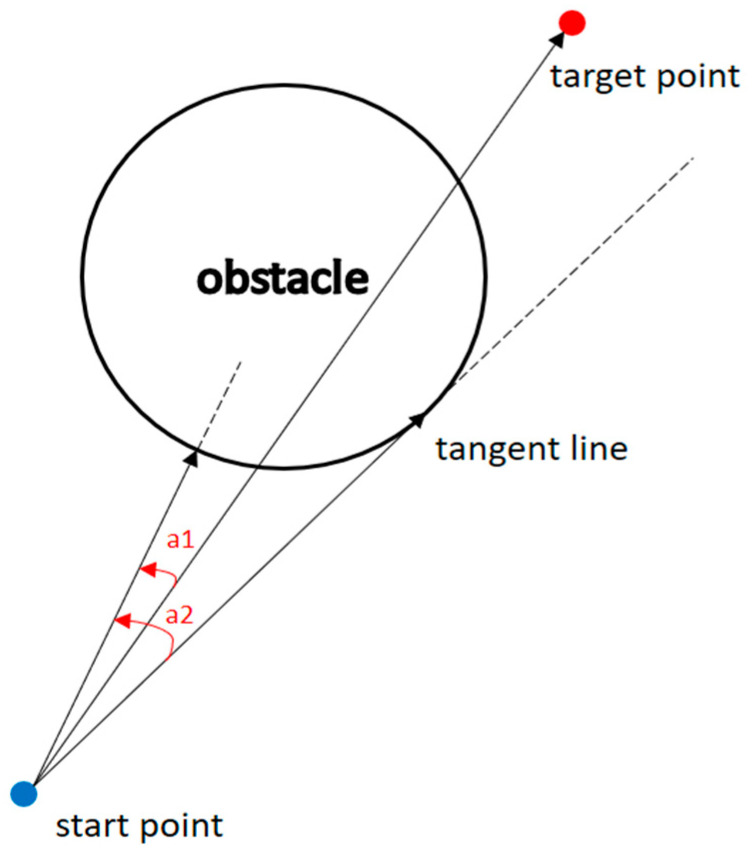
When the conditions are met.

If both conditions are met, the tangent can be used to calculate a new route, allowing the algorithm to generate a shorter path to avoid obstacles. Figure 8 shows that if the two conditions are met, the distance from the starting point to the target point is also greater than the distance from the starting point to the center of the obstacle when the angle between the obstacle and the target point (a1) is smaller than the angle between the obstacle and the tangent (a2). Obstacles were represented along the way so the researchers could use the tangents to help calculate the paths that generate shorter paths [25].

**Figure 9:**
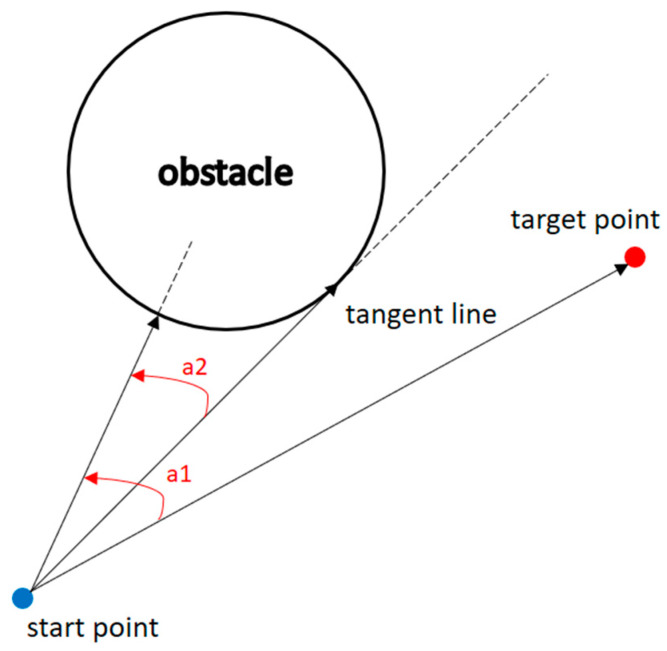
When the condition is not met.

As shown in Figure 9, the angle between the obstacle and the target point is greater than the angle between the obstacle and the tangent, indicating that the condition was not satisfied and that there is no obstacle on the path to the target.

(7) Fitness Function

Using the methods described in the previous sections, a new particle swarm *x* will be generated, and its fitness value will be calculated, as shown in the following formula:

(16) $$minf\left(x_{i}\right)=\left(\sqrt{{\left(g_{1}-x_{1}\right)}^{2}+{\left(g_{2}-x_{2}\right)}^{2}}\right)+\frac{1000}{\sqrt{{\left({obs}_{1}-x_{1}\right)}^{2}+{\left({obs}_{2}-x_{2}\right)}^{2}}}.$$

Here, $x_{i}$ is the *i*-th particle of particle swarm *x*, ($g_{1}$, $g_{2}$) is the coordinate of the target point, ($x_{1}$, $x_{2}$) is the particle’s current position, and (${obs}_{1}$, ${obs}_{2}$) is the coordinate of the nearest obstacle to the UAV. The smallest particle fitness value must be obtained, and the iteration must continue until the maximum number of iterations is reached.

(8) Fuzzy IPSO

**Figure 10:**
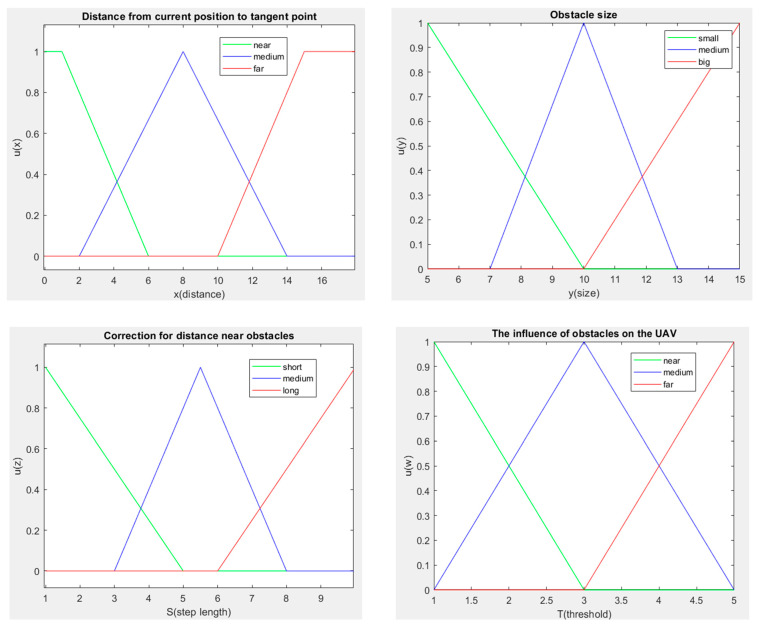
Fuzzy sets.

Due to the small number of obstacles encountered in this study, we used a fuzzy system to adjust the avoidance distance to obstacles and the maximum step size. Figure 10 shows the fuzzy sets, and the fuzzy rules are given as follows:

- $R^{1}:$ if *x* is near and *y* is small, then *S* is short and *T* is near.
- $R^{2}:$ if *x* is near and *y* is medium, then *S* is short and *T* is medium.
- $R^{3}:$ if *x* is near and *y* is big, then *S* is medium and *T* is far.
- $R^{4}:$ if *x* is medium and *y* is small, then *S* is medium and *T* is near.
- $R^{5}:$ if *x* is medium and *y* is medium, then *S* is medium and *T* is medium.
- $R^{6}:$ if *x* is medium and *y* is big, then *S* is long and *T* is far.
- $R^{7}:$ if *x* is far and *y* is small, then *S* is long and *T* is near.
- $R^{8}:$ if *x* is far and *y* is medium, then *S* is long and *T* is medium.
- $R^{9}:$ if *x* is far and *y* is big, then *S* is long and *T* is far.

*x* is the distance from the current position to the tangent point, and *y* is the size of the detected obstacle. *S* is the distance from the current path node to the next node; it is the UAV moving distance from the current location to the following location in each computing iteration. *T* is the influence of the obstacle on the UAV; it is the safety distance between the UAV and the obstacle. The values of the distance are the defuzzified results obtained from the fuzzy computation. The equation of the defuzzification is shown in Equation (17).

The defuzzification formula is as follows:

(17) $$y^{*}=\frac{\sum_{j=1}^{J}y^{j}\mu_{c^{i}}(y^{i})}{\sum_{j=1}^{J}\mu_{c^{i}}(y^{i})}.$$

Here, $y^{j}$ is the center of *y* of the *j*-th fuzzy rule, where *y* is the value that makes $\mu_{c^{i}}(y)$ maxima. *J* is the number of fuzzy rules. The y^*^ is the defuzzification value of *y*.

**Figure 11:**
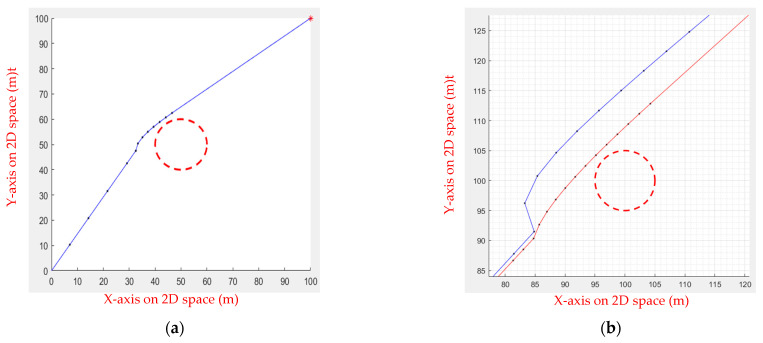
The flight path of obstacle avoidance. The red circle is the obstacle and the red star is the goal point. (**a**) IPSO combined with a fuzzy system to adjust the path; (**b**) comparison of IPSO (blue line) and Fuzzy IPSO (red line).

When the UAV approaches an obstacle that must be avoided, the UAV can start correcting the path earlier. As the UAV travels around the side and back of the obstacle, it gradually shortens the distance to the obstacle to a safe distance. The Fuzzy IPSO adjusts the length of the step based on the size and distance of the obstacle, avoiding extra path distance caused by the excessive step length (as shown in Figure 11), where the starting point is at (0, 0), and the ending point is at (100, 100).

(9) Dynamic Obstacle Avoidance

There are several methods for obstacle avoidance. Chengfu Jiang et al. improved the Artificial Potential Field method in 2021 [26]. In this study, the Artificial Potential Field method was applied to the real-time dynamic obstacle avoidance of UAVs so that UAVs could respond better when encountering unknown obstacles while performing tasks on a planned path [27].

1. Attractive Field

In the Artificial Potential Field (APF) method, attraction is the parameter used to find the target point. The attraction is typically proportional to the distance from the target point; the greater the attraction, the farther the distance from the target. To avoid too much attraction, the attraction gain is often used to adjust the attraction, as shown in the following formula:

(18) $$F_{att}=K_{att}\times \left(\left|{goal}_{x}-{pos}_{x}\right|,\left|{goal}_{y}-{pos}_{y}\right|\right).$$

Here, $K_{att}$ is the attraction gain and is usually a constant, ${goal}_{x,y}$ is the target location, and ${pos}_{x,y}$ is the current position. The amount of movement in the *X* and *Y* directions can be obtained by subtracting the current position from the target position and multiplying it by the attraction gain.

2. Repulsion Field

The device will be repelled by the obstacle once it enters the obstacle’s influence range. The repulsive force is inversely proportional to the distance from the obstacle, suggesting that the closer it is to the obstacle, the greater the repulsive force. To avoid the algorithm falling into the local optimal solution when calculating the repulsion force, this study added a formula for calculating the minimum repulsion force. The formula is given as follows:

(19) $$\left\{\begin{array}{c} F_{rep1}=K_{rep}\times \left(\frac{1}{rou}-\frac{1}{eff}\right)\times \frac{1}{{rou}^{2}}\times {dist}_{factor}\times \frac{\left(\left|{obs}_{x}-{pos}_{x}\right|,\left|{obs}_{y}-{pos}_{y}\right|\right)}{rou}\\ F_{rep2}=-K_{rep}\times {\left(\frac{1}{rou}-\frac{1}{eff}\right)}^{2}\times {dist}_{factor}\times {diff}_{dist}\; \end{array}\right..$$

(20) $$F_{rep}=F_{rep1}+F_{rep2}.$$

Here, ${Frep}_{1}$ is a small repulsive force that allows the algorithm to avoid a localized optimal solution. ${Frep}_{2}$ is the repulsion force of the obstacles, which allows the device to smoothly avoid obstacles. $K_{rep}$ is the repulsion gain, which is a constant and is used to adjust the size of the repulsion. *rou* is the horizontal distance between the device and the obstacle, *eff* is the influence range of the obstacle, ${obs}_{x,y}$ is the obstacle position, ${pos}_{x,y}$ is the current position, and ${goal}_{x,y}$ is the target location. ${dist}_{factor}$ and ${diff}_{dist}$ are given as follows:

(21) $${dist}_{factor}=\left|{goal}_{x}-{pos}_{x}\right|+\left|{goal}_{y}-{pos}_{y}\right|.$$

(22) $${diff}_{dist}=\left[\begin{array}{c} \left|{goal}_{x}-{pos}_{x}\right|\\ \left|{goal}_{y}-{pos}_{y}\right| \end{array}\right].$$

3. Total Potential Field

The total potential field is the resultant force of all obstacle’s attractive and repulsive potential fields. In the formula below, we quantified the displacement as 1 and added the *V* parameter to adjust the UAV’s flight speed:

(23) $$F=V\times {(F}_{att}+F_{rep})/|{(F}_{att_{x}}+F_{rep_{x}}),{(F}_{att_{y}}+F_{rep_{y}})|.$$

4. Artificial Potential Field Problem

**Figure 12:**
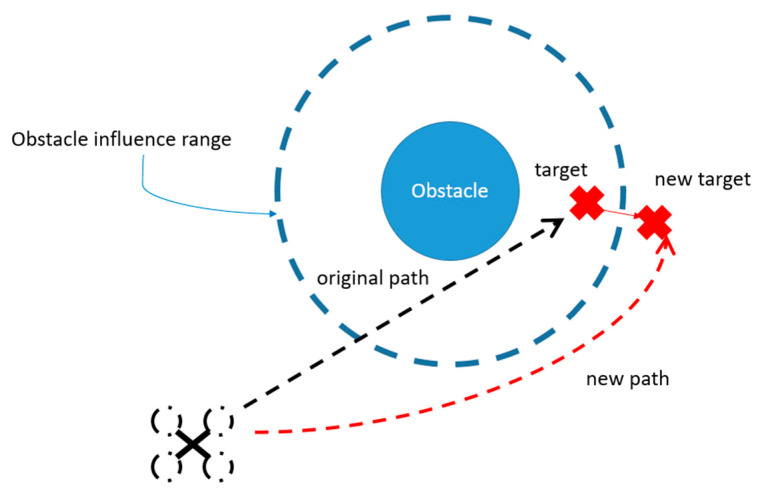
The target point being within the obstacle’s influence range.

Aside from the issue of falling into the local optimal solution, the artificial potential field may encounter a situation in which obstacles appear at the target point, preventing the device from approaching the target point, as shown in Figure 12. When this happens, the drone will move to a new target point closest to the target point but not within the obstacle’s sphere of influence and land.

## 5. Results

When the UAV performs the task, known and unknown obstacles must be considered, and the obstacles are classified as static and dynamic. This study classified dynamic obstacles as unknown obstacles and used APF for obstacle avoidance calculations. Static and known obstacle path planning was obtained using Fuzzy IPSO. We assume the detected obstacles are circular. LiDAR can detect an obstacle’s size (width). From a UAV point of view, the maximum size of the detected obstacle will be a circular shape; its diameter is the detected width. When the UAV encounters an unknown obstacle, the APF is used for obstacle avoidance, and the drone is commanded to follow the new calculated path. The APF calculates the avoidance direction and movement speed in real time. When obstacle avoidance is completed, the UAV will decide whether or not to continue path tracking. If there is an obstacle between the current position and the next waypoint, path tracking is continued, and the UAV will fly back to the original planned route. If there is no obstacle between the current position and the next waypoint, the UAV does not need to perform path tracking. Instead, the UAV can fly directly to the next waypoint. Several situations were simulated, and after ten operations, computational efficiency and path optimization of various algorithms in the following environments were compared.

(1) Large Static Obstacle

An obstacle with a radius of 60 was applied to test each algorithm’s path length and computing time when dealing with large obstacles. The operation is successful when the proposed system successfully guides the UAV to the end point. The process fails if it becomes trapped in a local optimum.

**Figure 13:**
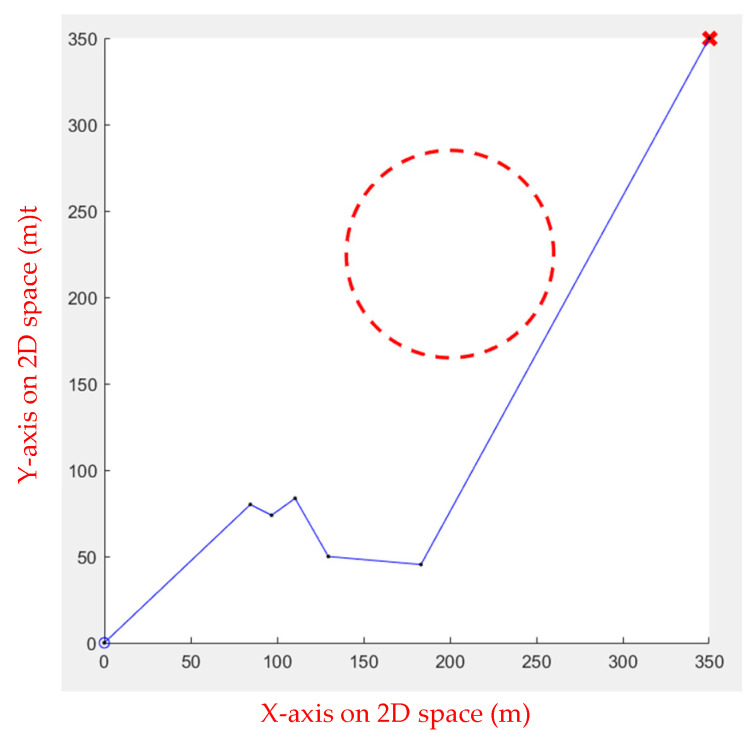
Genetic Algorithm for one obstacle.

Initially, the Genetic Algorithm was applied to the simulation environment. Although the Genetic Algorithm is an algorithm for finding the optimal solution, it is ineffective in use due to the need to adjust various parameters and adaptation functions in the algorithm based on the environment. Figure 13 shows the result, where the starting point is at (0, 0), and the goal point is at (350, 350).

**Figure 14:**
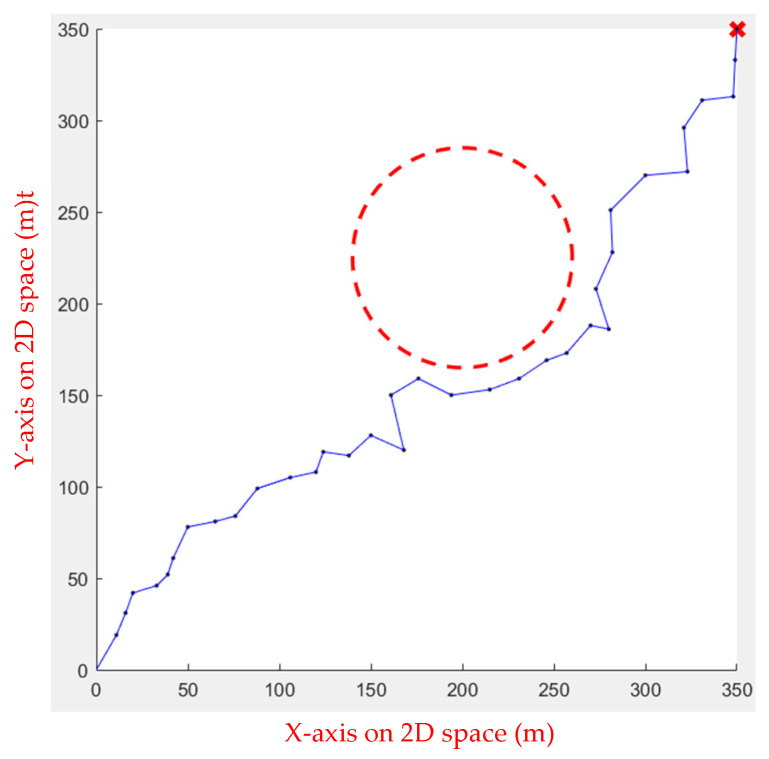
Ant Colony Algorithm for one obstacle.

The Ant Colony Algorithm was then applied in the simulation environment. The Ant Colony Algorithm is often used to solve the TSP problem, but it can also achieve good results for path planning. However, if the area of the environment is too large, the operation time is too long. Figure 14 illustrates the result.

**Figure 15:**
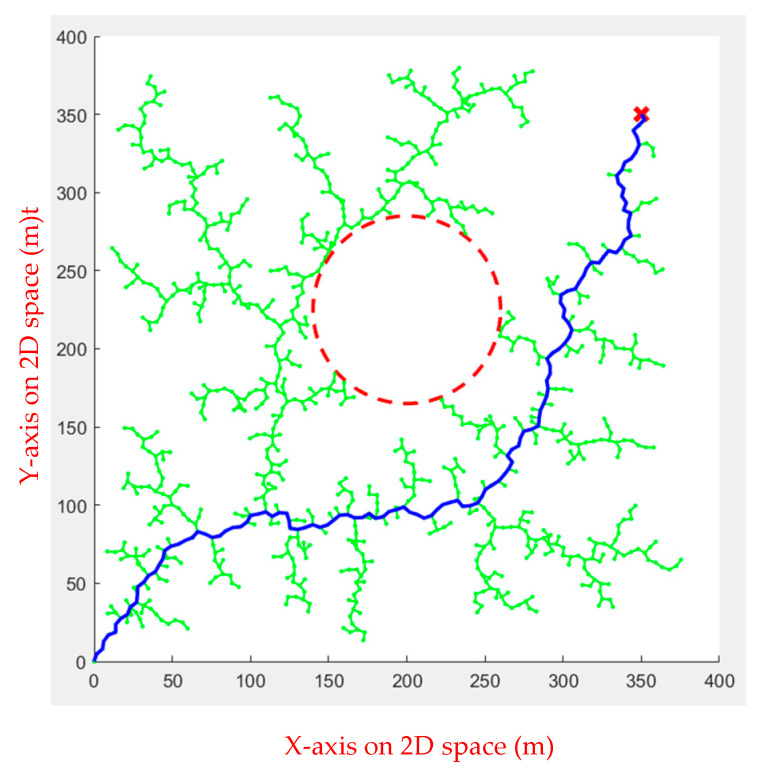
Rapidly Exploring Random Tree Algorithm for one obstacle.

Next, the Rapidly Exploring Random Tree Algorithm was applied to the simulation environment. Since the Rapidly Exploring Random Tree Algorithm, Ant Colony Algorithm, and Genetic Algorithm are heuristic algorithms, path planning fails. The success rate of the Rapidly Exploring Random Tree Algorithm is related to the execution time and the number of nodes. Figure 15 illustrates the result.

**Figure 16:**
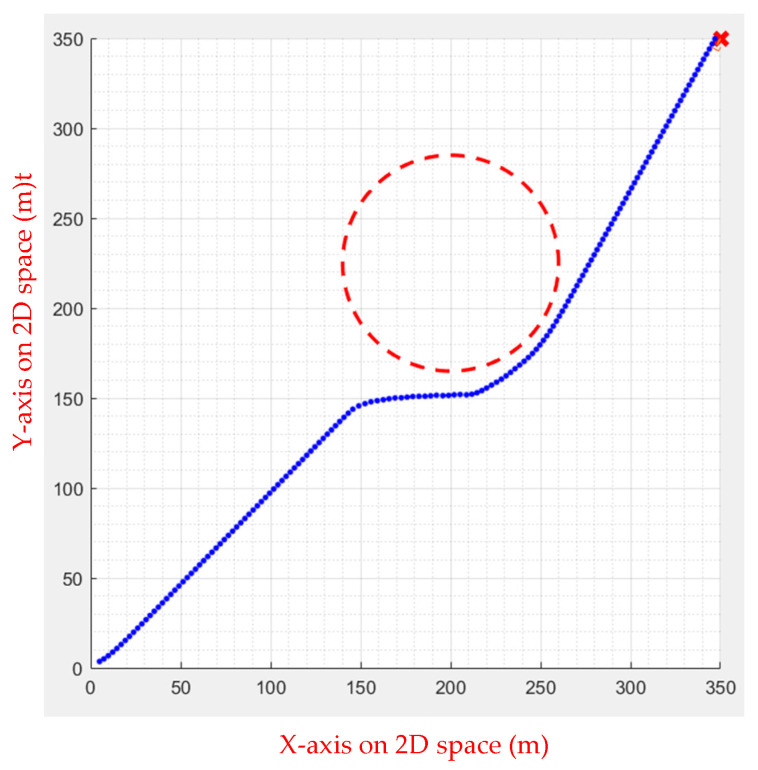
Artificial Potential Field for one obstacle.

Artificial Potential Field was then employed in the simulation environment. The result is shown in Figure 16.

**Figure 17:**
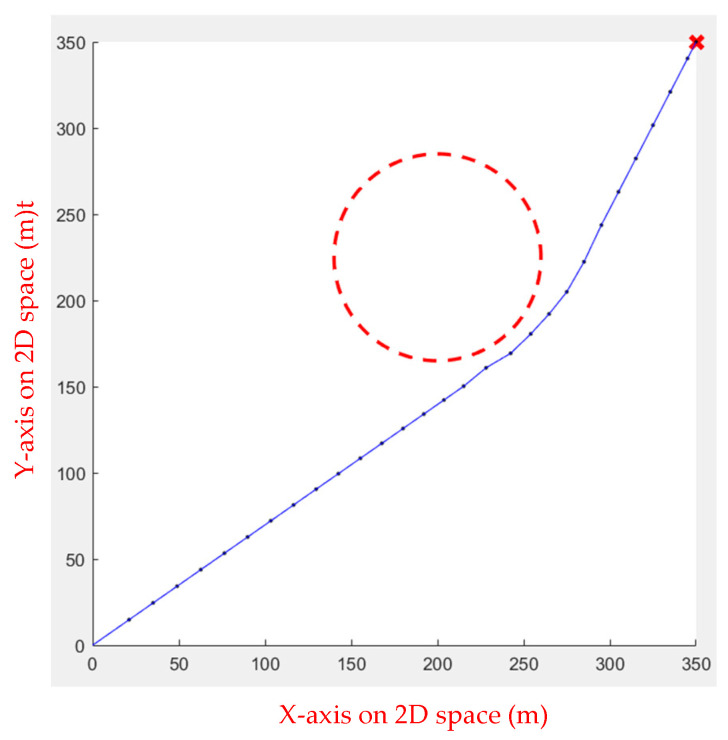
Improving the path using the first tangent.

**Figure 18:**
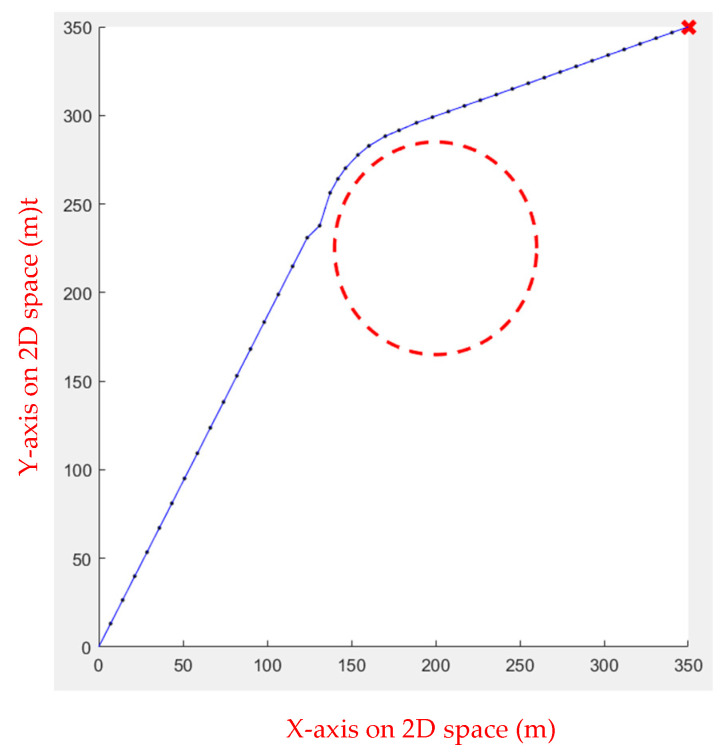
Improving the path using the second tangent.

Lastly, the proposed Fuzzy-Improved Particle Swarm Optimization (FIPSO) was applied to the simulation environment. Since an obstacle has two tangent points, the algorithm will first determine which path is better and keep it, as shown in Figure 17 and Figure 18.

Since the path generated by the first tangent is better, this study used the path improved by the first tangent. Table 2 illustrates the comparison results.

As seen in Table 3, each algorithm has undergone ten operations. Among them, the results of FIPSO and APF are the most consistent, with nearly identical values and paths each time. Although GA requires less operation time, the path’s instability makes GA a poor choice for path planning algorithms. The paths generated by ACO and RRT are also unstable, making the operation time too long.

(2) Multiple Waypoints

**Figure 19:**
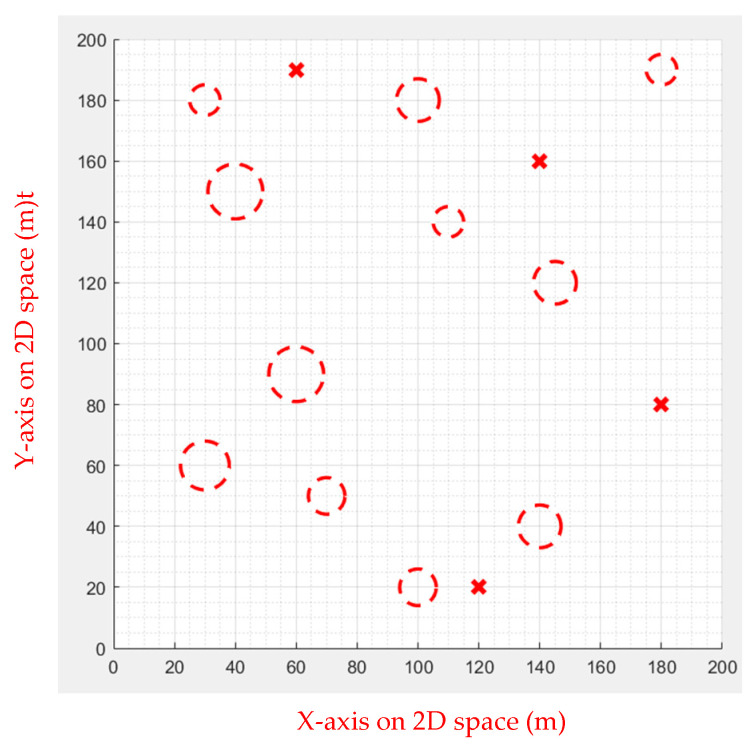
Environment 2, the red circles are the obstacles and the red x are the waypoints.

Multiple waypoints were given in this case, and the UAV must go through all the waypoints to the end. There will be obstacles in the cruise mission. Figure 19 shows the simulated environment.

**Figure 20:**
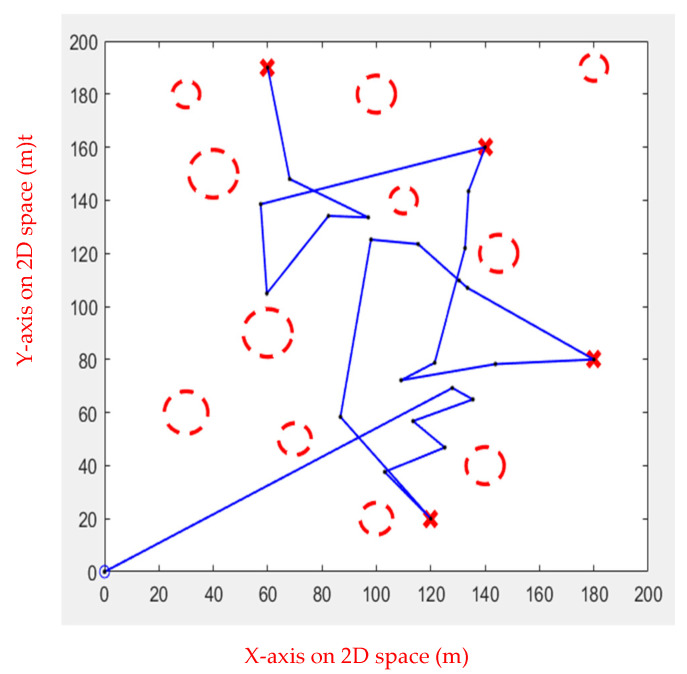
Genetic Algorithm for multiple obstacles.

**Figure 21:**
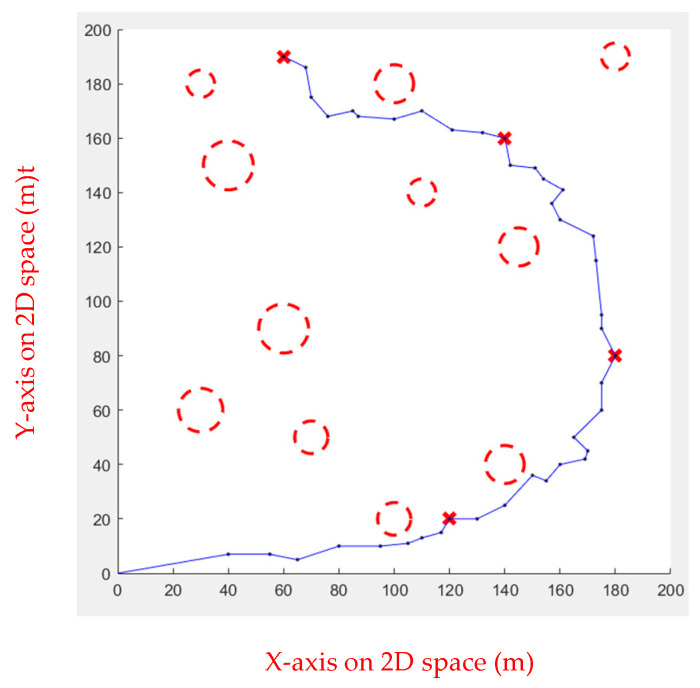
Ant Colony Algorithm for multiple obstacles.

**Figure 22:**
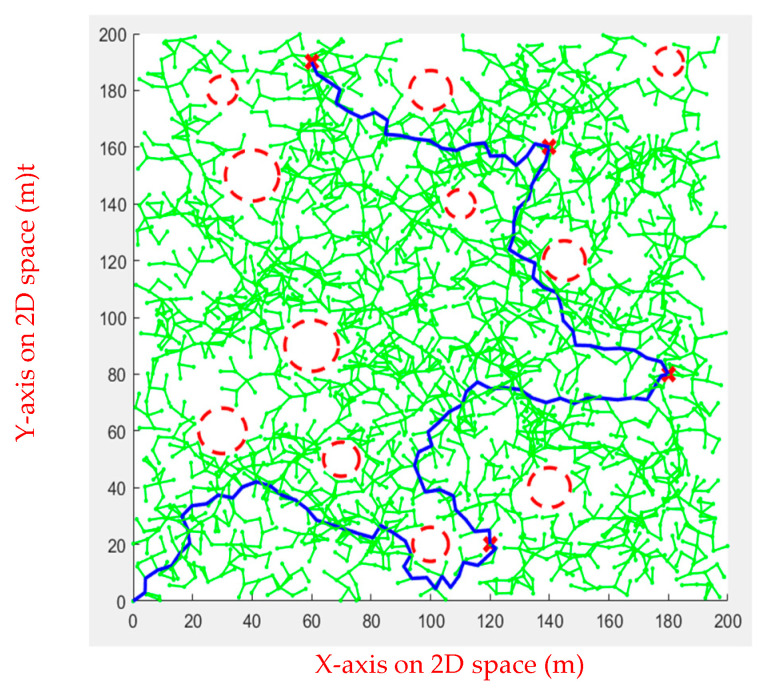
Rapidly Exploring Random Tree Algorithm for multiple obstacles.

**Figure 23:**
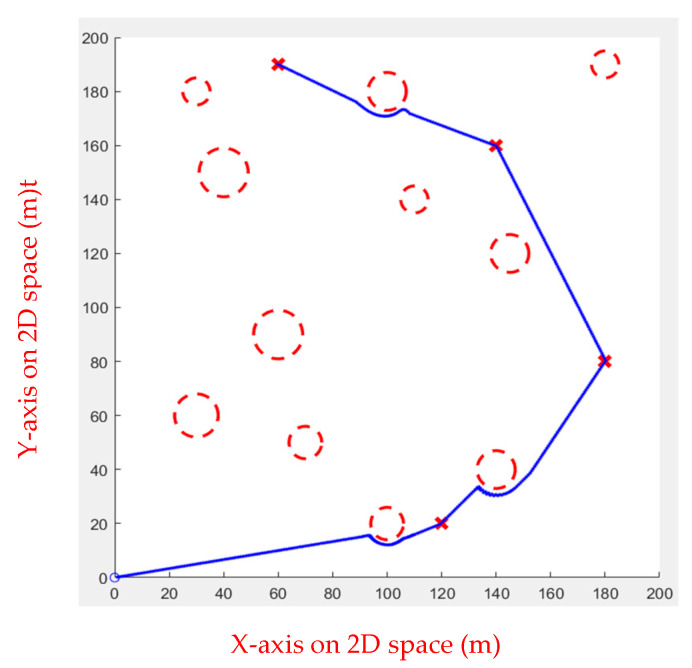
Artificial Potential Field for multiple obstacles.

In simulation Environment 2, each algorithm ran ten times, as shown in Figure 20, Figure 21, Figure 22 and Figure 23.

**Figure 24:**
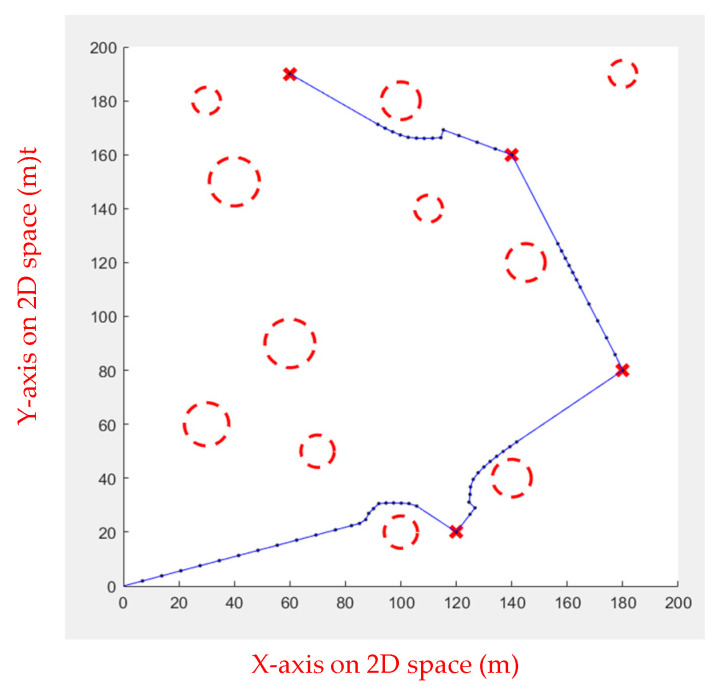
FIPSO first tangent.

**Figure 25:**
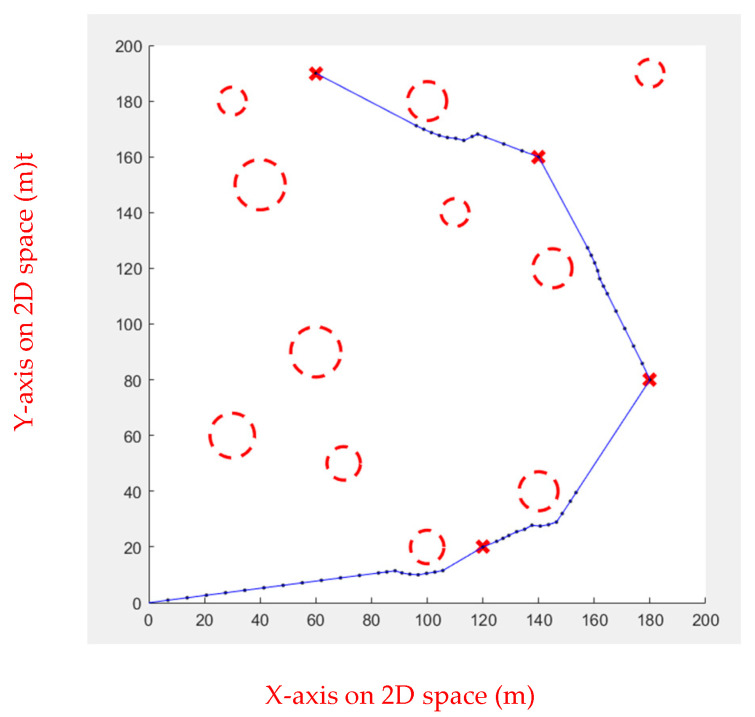
FIPSO second tangent.

As shown in Figure 24 and Figure 25, FIPSO calculates suitable tangent points for path planning and compares the path quality.

As can be seen from Table 4, the second tangent is better. Figure 25 illustrates the path.

Table 5 shows the performance of each algorithm in simulation Environment 2. It is apparent that in the simulated inspection environment, Fuzzy IPSO and APF perform well, but the APF performs slightly worse than Fuzzy IPSO. The other three have extremely unstable paths due to the randomness in Environment 2, resulting in poor performance.

(3) Dynamic and Static Environments

**Figure 26:**
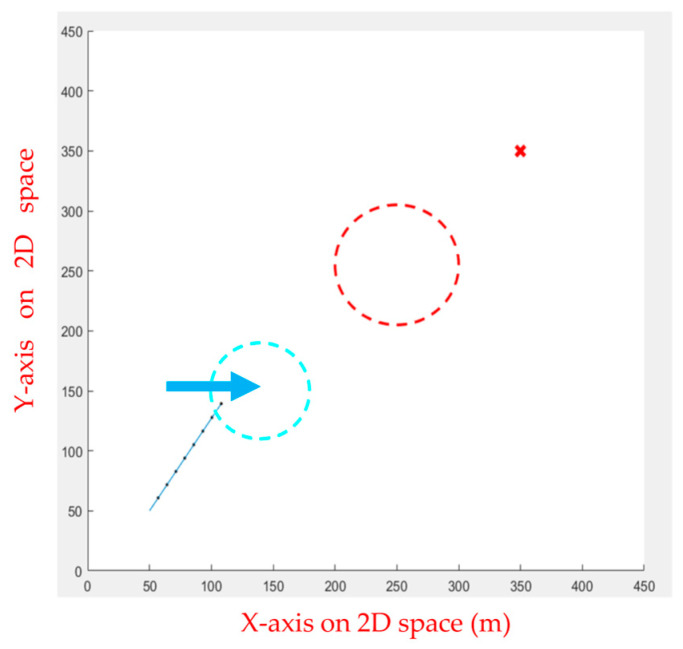
The blue circle is the moving obstacle, the blue arrow is the moving direction, the red circle is the static obstacle, and the red x is the goal point. The obstacle is moving too fast, causing the calculation to fail.

**Figure 27:**
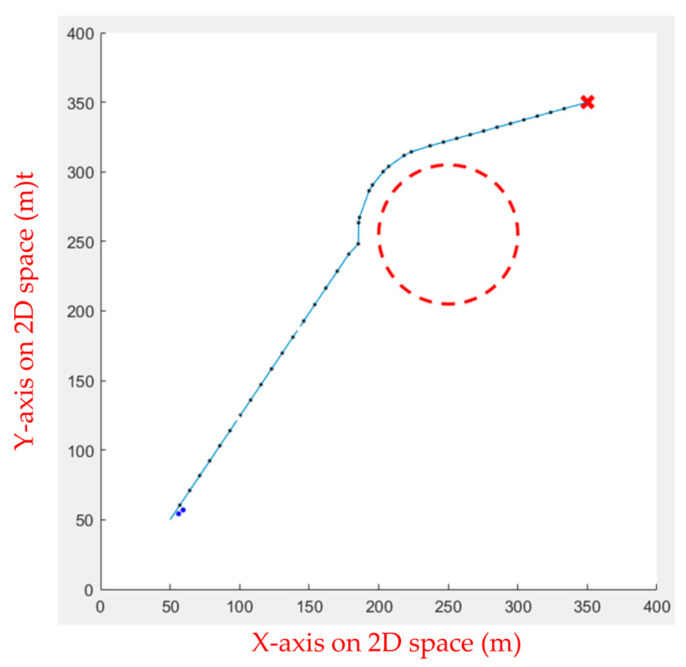
Path planning for static obstacles first.

**Figure 28:**
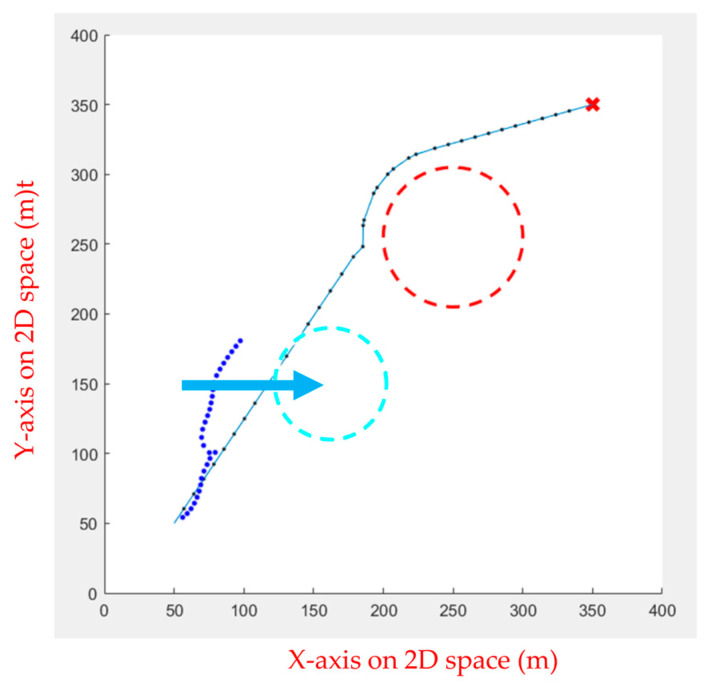
Obstacle avoidance using APF.

**Figure 29:**
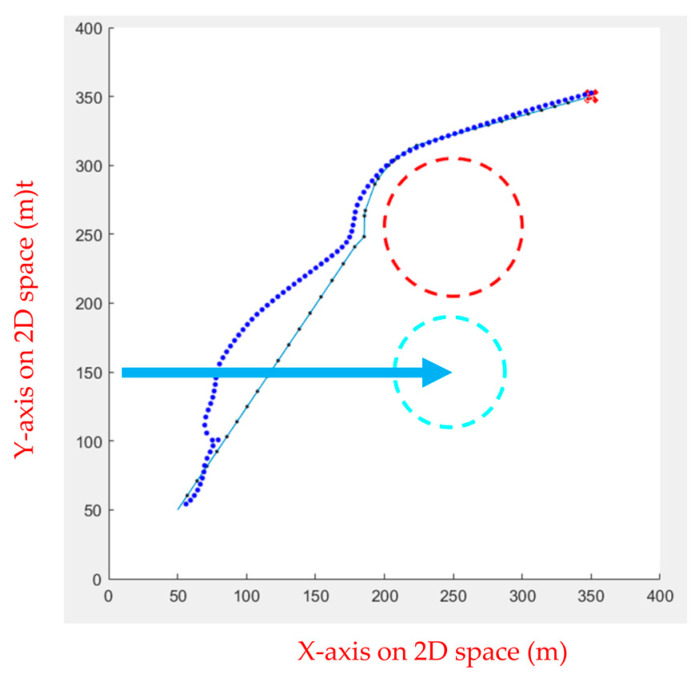
Continuing the path tracing and completing the mission.

**Figure 30:**
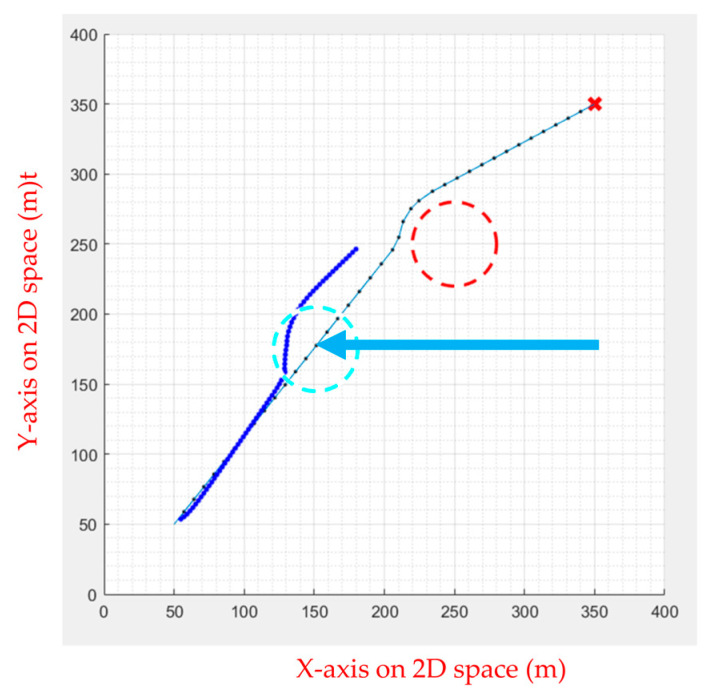
No contact with obstacles and no path tracing is performed.

**Figure 31:**
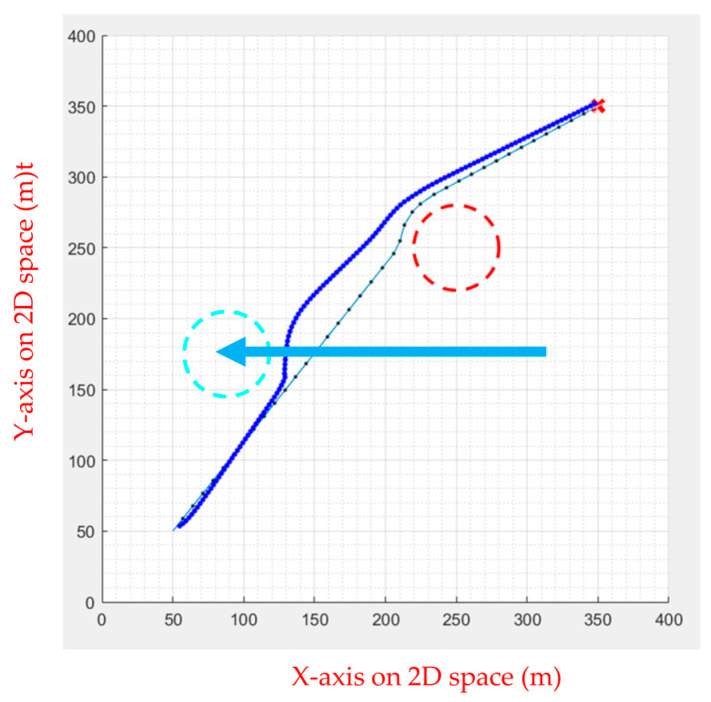
Final path.

FIPSO performs well compared to various path planning algorithms in the previous section, though the algorithm can also perform well when dealing with slow dynamic obstacles. However, obstacles the UAV encounters in the air are often faster, causing the path planning algorithm to fail in calculating the avoidance route, as illustrated in Figure 26. Therefore, the APF was used for dynamic obstacle avoidance, and the drone was commanded to follow the calculated path. When it encounters a moving obstacle, the APF calculates the avoidance direction and movement speed in real time, as shown in Figure 27, Figure 28, Figure 29, Figure 30 and Figure 31.

Initially, the FIPSO was used to plan the path with obstacles and find the best path, as shown in Figure 27.

Then, the APF was used for obstacle avoidance. When the UAV encounters unknown dynamic obstacles, it immediately avoids the obstacles, as shown in Figure 28.

When the obstacle avoidance is completed, the UAV will decide whether or not to continue the path tracking. If there is an obstacle from the current position to the end point, the path tracking is continued. As shown in Figure 29, the dotted line is the path generated using APF combined with path tracking, the dashed line is the optimal path for static obstacles generated by the FIPSO, and the arrow line is the moving direction of the dynamic obstacle.

Figure 30 and Figure 31 show no obstacle between the current position and the end point after avoiding an unknown dynamic obstacle, indicating that the UAV does not continue to perform path tracking. Instead, the UAV can fly directly to the end point.

(4) Campus Test

**Figure 32:**
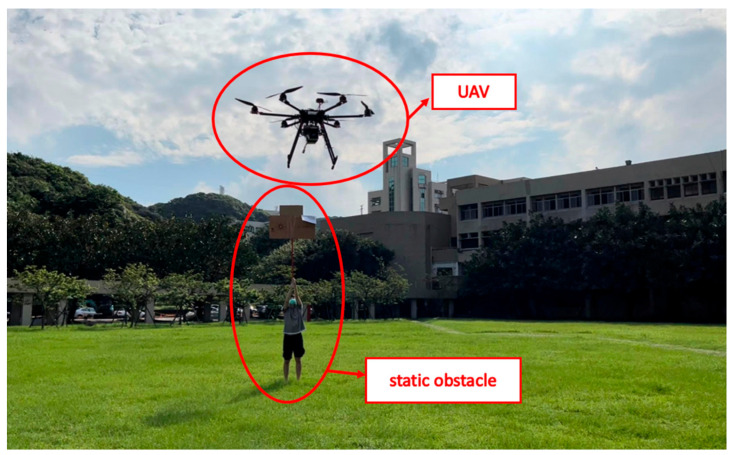
The UAV hovering in the air for path planning.

**Figure 33:**
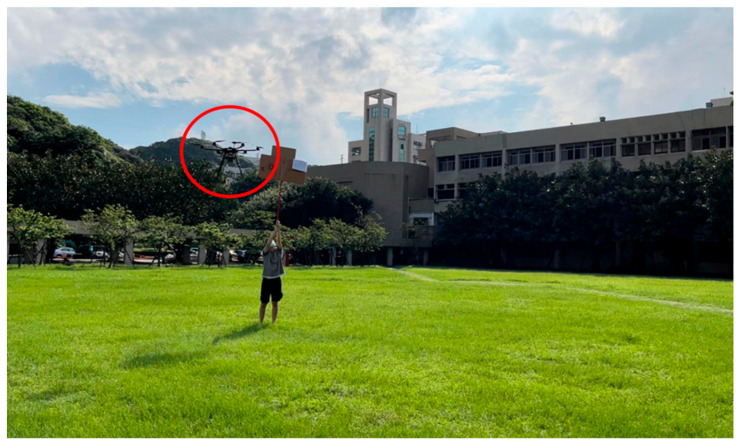
The UAV avoids static obstacles, the UAV is marked by the red circle.

**Figure 34:**
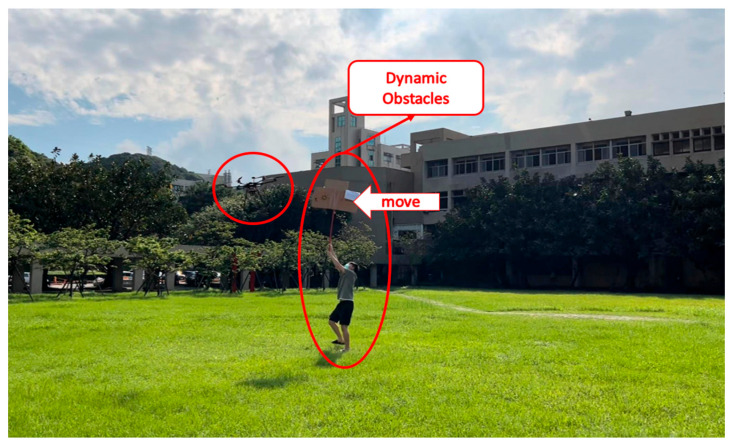
A dynamic obstacle moving to the left and interfering with the UAV.

**Figure 35:**
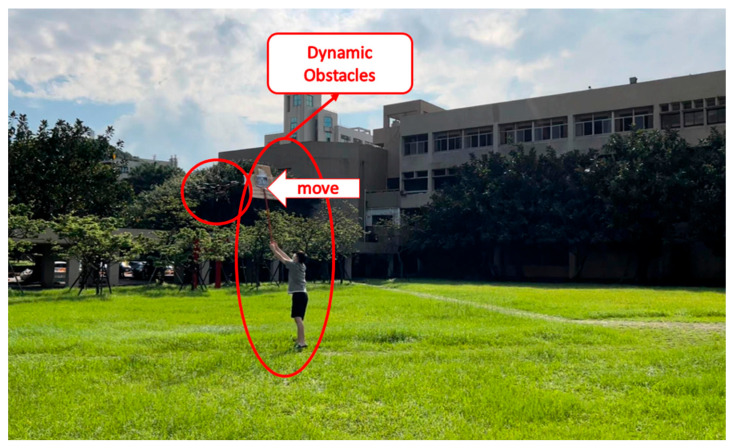
Sensing the second dynamic obstacle.

**Figure 36:**
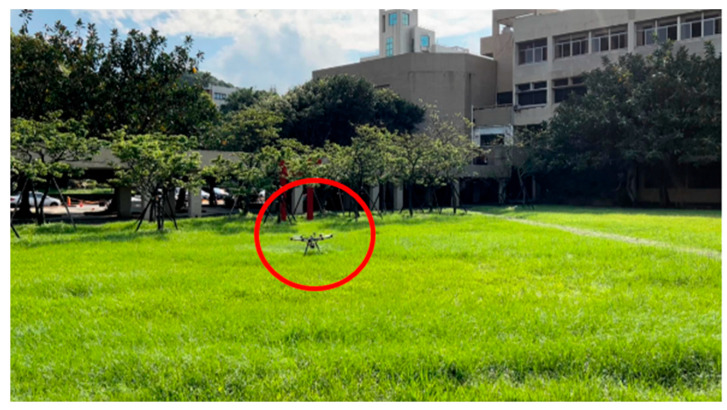
Reaching the destination.

**Figure 37:**
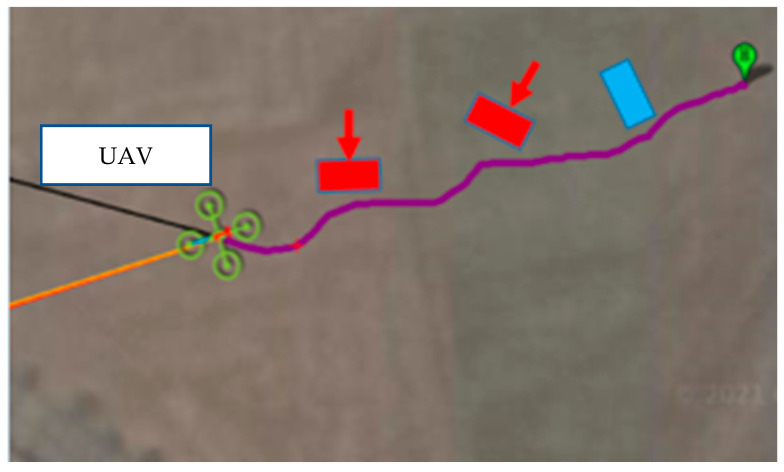
The obstacle avoidance path (purple line): the red parts are the dynamic obstacle, the red arrow is the moving direction, and the blue part is the static obstacle.

The field test was first conducted on campus. The tasks include path planning and obstacle avoidance. A river-side test was also performed. The UAV system first detected the obstacles in the air and planned a path. Then, the UAV performed path tracking. When the UAV encounters dynamic obstacles during flight, it can avoid them immediately. A target point was set, and the UAV encountered dynamic obstacles on the way to the target point, as shown in Figure 32, Figure 33, Figure 34, Figure 35, Figure 36 and Figure 37.

**Figure 38:**
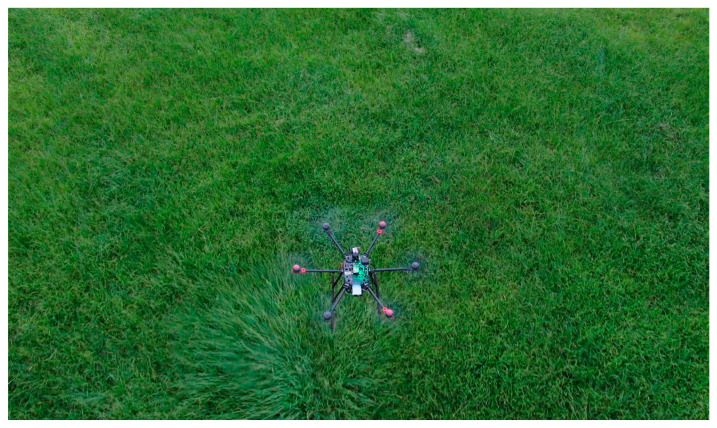
Flying to the first waypoint.

**Figure 39:**
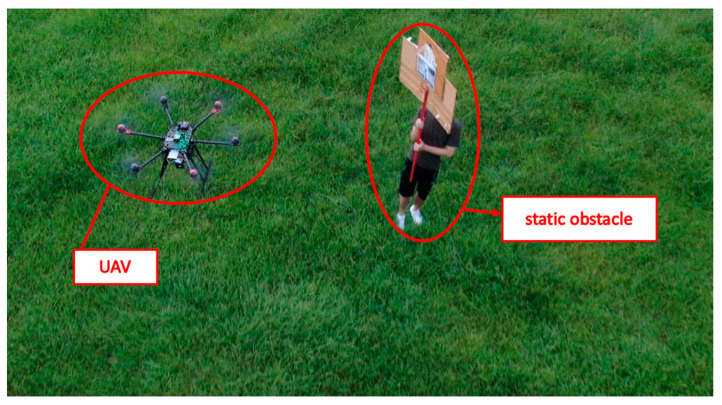
Avoiding static obstacles when heading to the second waypoint.

**Figure 40:**
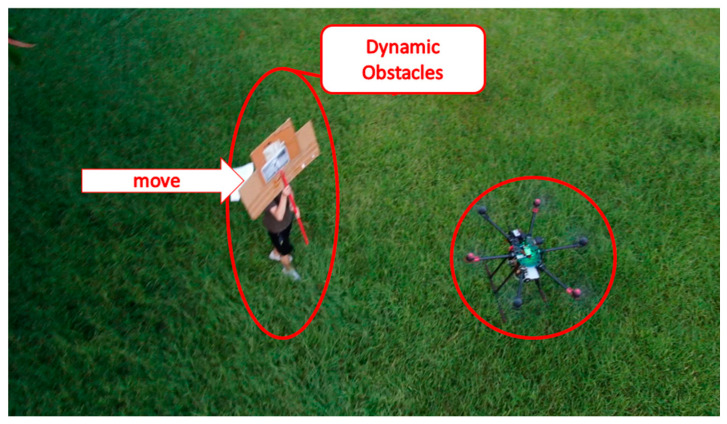
Dodging dynamic obstacles when heading to the end point.

**Figure 41:**
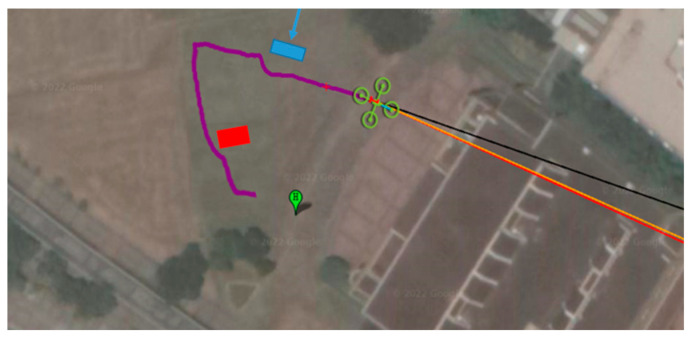
The flight path: the red part is the static obstacle, the blue part is the dynamic obstacle, and the white points are the waypoint and the end point.

In the second test, two waypoints and a destination were set. There were no obstructions on the way to the first waypoint. A static obstacle was present on the way to the second obstacle. Figure 38, Figure 39, Figure 40 and Figure 41 show a dynamic obstacle on the way to the end point.

(5) Riverbank Test

**Figure 42:**
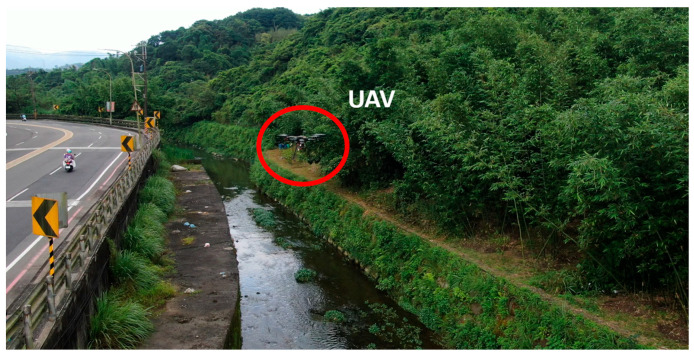
Side view of the UAV and the riverbank.

**Figure 43:**
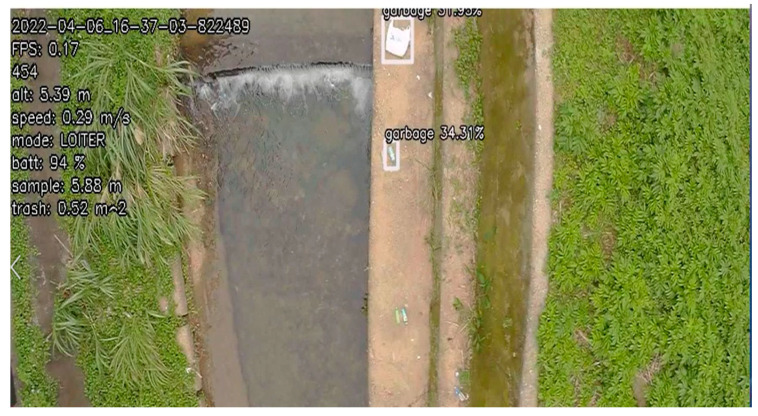
Garbage was detected via the on-board image recognition system.

**Figure 44:**
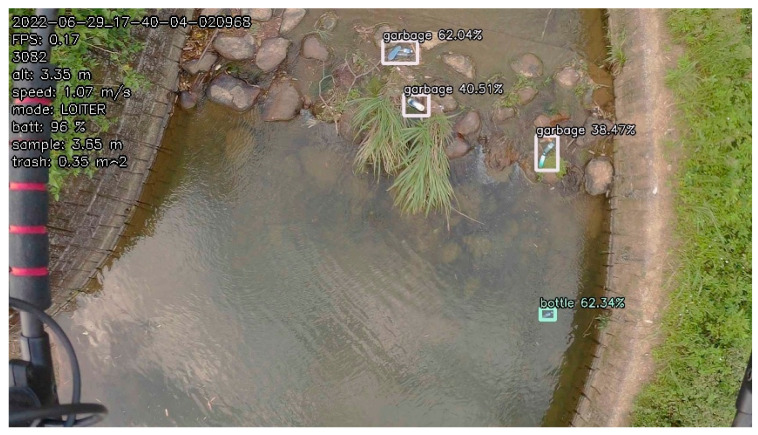
More garbage was detected.

**Figure 45:**
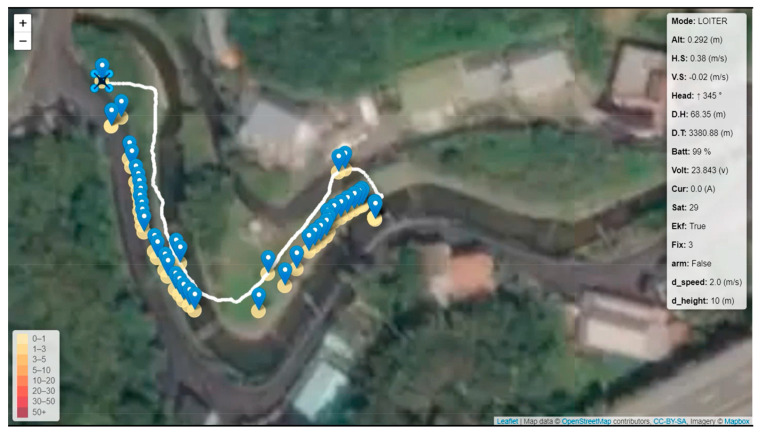
The flight path, waypoints, and garbage information.

First, the waypoints were planned according to Google Maps. Waypoints are the turning points of the river. Then, static obstacles were marked on the map manually. Finally, the path planning method was applied to obtain the optimal path. Then, the optimal path was embedded in the onboard computer with path coordinates. The Tienliao River (Keelung City, Taiwan) was the test field, as shown in Figure 42. During the riverbank inspection, garbage on the riverside was detected using an onboard image recognition system [28], as shown in Figure 43 and Figure 44. The flight path, waypoints, and garbage information were sent back to the ground base control center via WiFi, as shown in Figure 45. According to the results of the field tests, the proposed UAV system can perform riverbank inspection successfully.

## 6. Conclusions

This study focused on path planning and obstacle avoidance involved in automated riverbank inspection. The results suggested that the shorter the planned path, the less energy is required, making the inspection more efficient with a longer operating distance. In this study, the Improved Particle Swarm Optimization combined with a fuzzy system and system equations was proposed to handle UAV cruise tasks for fixed-point rivers. An effective avoidance action could be taken if an obstacle was detected on a flight path. The comparison results of the different path planning algorithms revealed that the proposed Fuzzy IPSO was more efficient for path planning than conventional methods, such as GA, ACO, RRT, and PSO. Fuzzy IPSO also outperformed the recently issued IPSO in path length and computation time. The proposed system was applied to inspect several rivers in Taiwan, which were the Tienliao River, Sieding River, and Keelung River. The UAV flew along the planned paths and avoid obstacles automatically. Most trash on the riverbank was identified, and the UAV system transmitted garbage information to the control center in real time. The proposed system could relieve the workforce in riverbank inspection and significantly reduce costs.

The UAV would sometimes shake up and down due to the strong wind during the field test on the river. However, if obstacles are above or below the UAV, the UAV is prone to crashing. More sensors can be added in the future so that the sensors can not only sense obstacles in the 2D plane but also detect obstacles in all 3D directions to increase the UAVs’ safety. In this study, it was assumed that the obstacles are mainly circular, although they often have different shapes in reality. Future research is suggested to consider more shapes when running simulations. This improvement allows the algorithm’s plan to be reduced even further. The most serious problem encountered in this study was the abnormal compass caused by magnetic field interference. When the UAV flies along a river, it will usually encounter bridges. Most bridges are reinforced concrete bridges; under certain conditions, the magnetic field generated by the steel bars may cause the UAV’s compass to malfunction, putting the UAV in danger of crashing. It is possible to prevent magnetic field interference by using a three-axis geomagnetic sensor in future works.

## Figures and Tables

**Table 1:** Comparison of the operation speed of the three inertia weights.

Execution Time (s)	Set *λ* = 0.5	Log Generation	Linear Generation
Environment one	6.274939	5.925654	5.876535
Environment two	18.913818	18.629813	18.357249

**Table 2:** Comparison of the first tangent with the second tangent for one obstacle.

	First Tangent	Second Tangent
Path length	507.4689	528.4383

**Table 3:** Comparison of each path planned in Environment 1.

	GA	ACO	RRT	APF	FIPSO
Length	593.5808	608.3265	656.0714	509.5011	507.4692
Time (s)	0.542633	8.035564	8.655947	8.623706	6.132645

**Table 4:** Comparison of the first tangent with the second tangent for multiple obstacles.

	First Tangent	Second Tangent
Path length	399.5292	389.7921

**Table 5:** Comparison of each path planned in Environment 2.

	GA	ACO	RRT	APF	FIPSO
Length	935.8862	503.2649	558.0614	396.2307	389.7919
Time (s)	6.147131	5.465801	6.312311	5.321153	3.454912

## Data Availability

Data are contained within the article.
